# Using non‐linear slide decks to administer individualized problem‐based learning assessments within pharmacology education

**DOI:** 10.1002/bcp.70248

**Published:** 2025-08-21

**Authors:** Wendy R. Francis, Nia A. Davies, Aidan Seeley

**Affiliations:** ^1^ Swansea University Medical School Swansea University UK

**Keywords:** assessment, education, pharmacology, problem‐based learning

## Abstract

**Aim:**

Problem‐based learning (PBL) is an established approach in medical, nursing, pharmacy and veterinary medicine education. This study describes the implementation and aims to evaluate the use of non‐linear slide decks as a method to deliver PBL as individualized student assessments within pharmacology education. This approach, originally developed in response to the COVID‐19 pandemic, has been evaluated over 5 years since its integration within undergraduate pharmacology modules.

**Methods:**

Two non‐linear slide decks were designed using Microsoft PowerPoint with interactive hyperlink navigation, allowing dynamic and student‐directed progression through two scenarios: a summative clinical toxicology assessment and a formative pharmacokinetic calculation activity.

**Results:**

Comparative analysis of student performance demonstrated that grades achieved via non‐linear PBL (67.6 ± 10.4%, n = 140) were comparable to those from essays (65.3 ± 12.7%, n = 83) and oral presentations (66.0 ± 6.5%, n = 56), though lower than online quizzes (71.4 ± 11.3%, n = 141). Furthermore, student feedback (n = 31) demonstrated positive student perceptions of non‐linear PBL.

**Conclusion:**

Despite the increased complexity involved in developing and integrating non‐linear PBL resources, this methodology offers an engaging, flexible and pedagogically robust strategy that promotes active learning. It addresses long‐standing challenges associated with group‐based PBL formats while meeting recommended pharmacology education curricula.

**What is already known about this subject**

- Problem‐based learning (PBL) is widely used in health education and is known to enhance knowledge acquisition and student satisfaction.
- Traditional PBL is group‐based, presenting challenges with individual assessment, communication and grading.
- COVID‐19 disrupted group learning formats, highlighting the need for adaptable, individually assessed PBL methodologies.

**What this study adds**

- Non‐linear slide decks are a viable method for delivering individualized PBL assessments.
- Student performance using non‐linear PBL is comparable to conventional assessment techniques, with positive student perceptions.
- This approach offers an engaging individualized alternative to group‐based PBL but is more complex to administer than conventional assessments.

## 1 INTRODUCTION

Problem‐based learning (PBL) is a pedagogical approach first employed by McMaster University in the 1960s.^1^ Since then, PBL has become an established approach in medical, nursing, pharmacy and veterinary medicine education.^2^, ^3^, ^4^, ^5^ While traditional assessment methodologies focus on retention of didactic teaching content rather than its application to real‐life scenarios, case‐based learning (CBL) uses real‐world clinical cases and can be incorporated into pharmacology education to facilitate the practical application of pre‐existing knowledge to improve comprehension.^6^

PBL, however, provides an opportunity to facilitate deeper understanding where students have more autonomy and flexibility in the learning process to explore complex problems and identify practical solutions.^7^ PBL has been shown to improve learning and knowledge acquisition^8^, ^9^, ^10^, ^11^ while also being positively received by students.^5^ Whilst studies of gender differences in academic performance within pharmacology education are conflicting,^12^, ^13^ PBL in pharmacology education has been shown to be effective for diverse learners regardless of gender.^14^, ^15^

PBL, traditionally, focuses on students working in small groups to identify and address a problem with the aim being to replicate realistic problems to promote student integration of theory and practice through the application of knowledge and skills.^16^ However, group work provides its own challenges as it is often negatively received by students due to challenges with group communication and scheduling meetings, as well as summative assessments that often do not allow for students to be graded individually.^17^, ^18^

Furthermore, the COVID‐19 pandemic had significant impacts on the ability to conduct group work due to social distancing, virtual contexts and increased external distractions.^19^ The COVID‐19 pandemic had a significant impact on higher education sectors globally, with many education providers having to adapt their teaching and learning practices by pivoting to online platforms,^20^ including the delivery of PBL sessions.^21^

**FIGURE 1:**
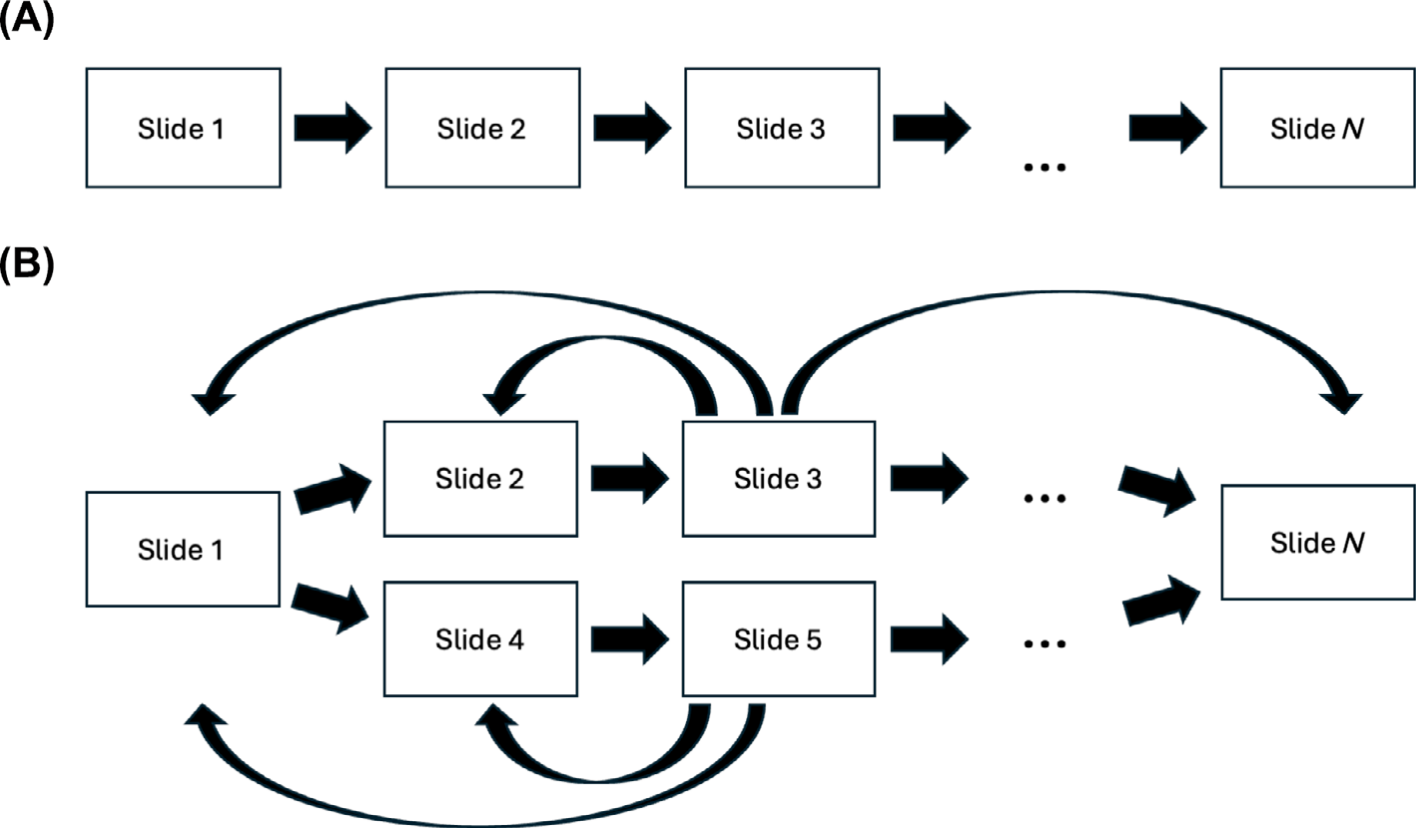
Linear presentations and non‐linear slide decks (A) representative schematic of a typical linear presentation, which progresses sequentially from slide 1 to slide *N*. (B) Representative schematic of a non‐linear slide deck, which does not follow a single path, whereby students can select to move between content from slide 1 to slide *N*.

Here, we describe the use of interactive non‐linear slide decks to administer individualized formative and summative PBL assessments (hereafter referred to as non‐linear PBL), initially developed as an alternative methodology during the COVID‐19 pandemic. While standard linear presentations have structured, sequential content which progress in a predefined order, moving from slide 1, slide 2 to slide *N* (Figure 1A), non‐linear slide decks does not follow a single predefined path (Figure 1B); students can select *how* to move between content from slide 1 to slide *N*, allowing dynamic navigation through the content.^22^ Whilst linear PBL can limit a student's ability to explore diverse approaches to problem solving in complex scenarios, non‐linear PBL offers the flexibility to explore multiple pathways and branching scenarios to find solutions to problems.^23^ This can promote deeper understanding and higher levels of critical thinking, both at a formative and summative level and can act as a good feedback tool for learning.^24^, ^25^

In this study, non‐linear PBL assessments were conducted individually by students while retaining the goals of conventional PBL, to develop flexible student knowledge and effective problem‐solving skills,^26^ with two non‐linear PBL slide decks prepared.

The first integrates clinical toxicology and patient presentation following a poisoning event, with this topic being timely when training in toxicology has declined^27^ and the identification of toxicology as a knowledge and skills gap at degree level.^28^

The second, administered as a formative assessment, focuses on pharmacokinetic calculations, which has been identified as a consistent skills shortage in UK graduates.^29^, ^30^ Furthermore, both topics are identified within the British Pharmacological Society's undergraduate core curriculum as core knowledge requirements within undergraduate pharmacology degrees.^31^

In this study, we evaluate the use of non‐linear PBL over 5 years since its integration as an alternative methodology during the COVID‐19 pandemic compared to standard assessment methods, and we evaluate the grades achieved by male and female students to identify if this novel approach is impacted by gender, and outline the challenges and advantages of this teaching methodology.

## 2 METHODS

**FIGURE 2:**
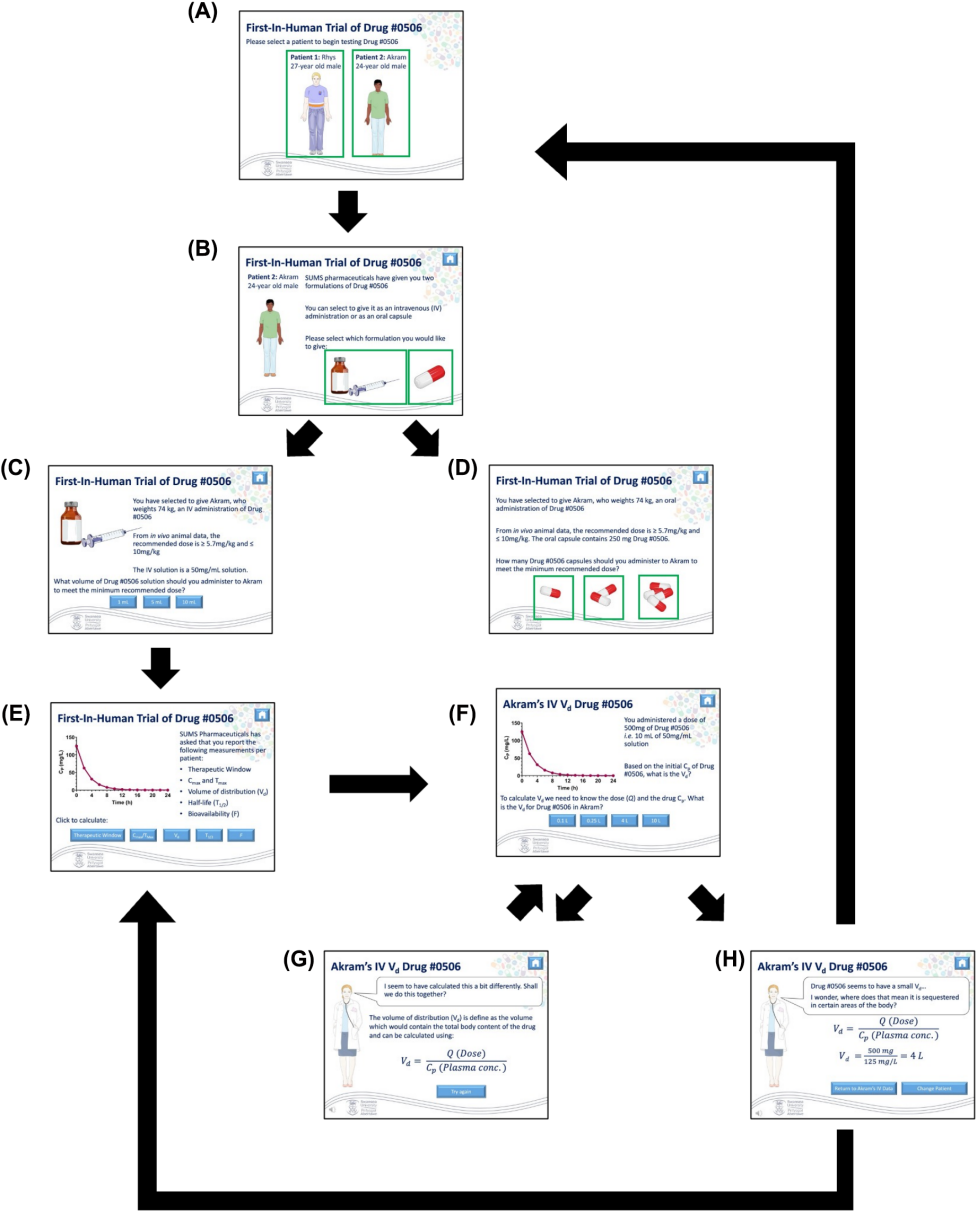
Exemplary slides used for formative non‐linear PBL used for pharmacokinetic calculations. (A) Branching options allow students to select which patient they wish to review and (B) select either intravenous or oral administration of the fictitious drug #0506. Students are then directed to calculate the correct dosage for either (C) intravenous or (D) oral administration. (E) Once the correct dosage is selected, students are directed to select which pharmacokinetic parameter to calculate (therapeutic window, *C*_max_, *T*_max_, *V*_d_, *T*_1/2_, *F*). (F) Exemplary slides for calculation of drug #0506 *V*_d_, which (G) if calculated incorrectly will provide immediate feedback and then prompt student to retry by returning to (F) or (H) if calculated correctly will allow them to return to (A) to change their patient or return to (E) to review another pharmacokinetic parameter. Hotspot selection images, where users can click on specific areas to take an action, are highlighted in green, and hyperlinked buttons for slide progression are shown in blue. Artwork from Servier medical art, licensed under CC BY 4.0.

To facilitate flexible, student‐directed navigation through the slide decks, we developed two non‐linear Microsoft PowerPoint slide decks using internal hyperlink functionality. Using PowerPoint's “Insert > Link > Place in This Document” functionality, navigation through the slide deck was controlled through either hotspot selection images, where users can click on specific areas to take an action, or hyperlinked buttons to sections in the slide deck (Figure 2). This enabled non‐linear navigation directly to relevant slides (Figure 2A,B), enabled branching based on student selections (Figure 2B‐D) or facilitated backwards navigation (Figure 2F,G). ‘On Mouse Click’ transitions were disabled to prevent linear navigation through the slide deck, and each hyperlink was verified to ensure accurate slide referencing. Slide decks were tested for both Windows and macOS operating systems to confirm cross‐platform functionality. Non‐linear PBL assessments were distributed by embedding the slide deck within the module virtual learning environment (VLE) using Microsoft PowerPoint's “Share > Embed this presentation” functionality and packaged with instructions for use and navigation within the VLE.

The first non‐linear PBL was a summative assessment based on clinical toxicology and patient presentation following a poisoning event. This was administered within a compulsory 20‐credit pharmacology and toxicology module to students in their second year of a 3‐year BSc Medical Pharmacology course at Swansea University. The non‐linear PBL was administered as a summative assessment following seven 1‐h lectures on patient presentation and clinical strategy for the toxicity of alcohols, over‐the‐counter medications and drugs of abuse.

The non‐linear PBL presents a fictitious patient, Mr E, who was admitted to hospital following a poisoning event. Students assume the role of a clinical toxicologist during the non‐linear PBL and are required to identify the primary cause of the patient's toxicity. Students are provided with the patient's vital signs, and they can select additional tests through hyperlinked buttons, including blood gas analysis, urine dipstick tests and urine and/or blood tests for chemicals, over‐the‐counter medications and illicit substances. For urine and/or blood tests, students had the option to select a qualitative test, providing a positive/present or negative result, and/or a quantitative test, where the test result had a numerical result. Quantitative tests were accompanied by levels of toxicity, based on clinical guidelines from the National Poisons Information Service, ranging from none to potentially fatal. Students could have, depending on how they navigate through the non‐linear PBL, identified negative results to rule this out as the cause of toxicity or identified positive results for compounds at sub‐toxic levels that were not the cause of the patient's toxicity and clinical presentation. Irrespective of the route the student selected, only one compound would be the primary cause of toxicity, but this compound was changed for resubmission attempts.

On identification of the primary cause of toxicity, students were then required to complete a short toxicology report based on the results obtained and how this led to their conclusion of the primary cause of toxicity, and students had to address short answer questions (SAQs) based on their findings from the non‐linear PBL.

Students enrolled on this module required an overall grade of ≥40% to pass the module. The overall grade was calculated from three assessments: (i) an online quiz containing both multiple choice questions (MCQs) and SAQs, for 30% of the overall module mark, (ii) an essay‐based assessment (utilized from 2019/2020‐2021/2022) or oral presentation (utilized from 2022/2023‐2023/2024) for 30% of the module mark and (iii) the non‐linear PBL, which accounted for 40% of the module mark. The pass mark for each assessment component was 40%.

To evaluate the use of summative non‐linear PBL assessments, grades, scored as a percentage, were compared to the grades achieved within this module for the online quiz (2019/2020‐2023/2024), the essay‐based assessment from three academic years (2019/2020‐2021/2022), an oral presentation assessment from two academic years (2022/2023‐2023/2024) and the grades for the summative non‐linear PBL assessment from five academic years (2019/2020‐2023/2024). Assessment grades and gender data were obtained from institutional records.

Zero scores arising from non‐submission of assessments were excluded from the assessment grade and gender analysis. Zero scores were included in overall module grade calculations. Comparison of assessment grades were analysed by two‐way ANOVA with Tukey's post‐test compared to non‐linear PBL grades. Grades achieved by gender across 5 years using non‐linear PBL were analysed by two‐way ANOVA with Bonferroni post‐test. Overall module grades were analysed by the Kruskal‐Wallis test with Dunn's post‐test. Statistical significance was set at *P* < 0.05. All analyses were performed using GraphPad Prism 10.

Qualitative feedback on summative non‐linear PBL was extracted from student e‐mail correspondence and obtained from institutional module feedback records.

The second non‐linear PBL was a formative assessment based on pharmacokinetic calculations, enabling students to practise these calculations and receive immediate feedback on their answers. This was administered to students enrolled on BSc Medical Pharmacology or BSc Applied Medical Sciences within a 20‐credit pharmacology module during their final year of a 3‐year course at Swansea University as an optional resource for consolidation of lecture materials following six 1‐h lectures on pharmacokinetic calculations.

The non‐linear PBL contained exemplary data based on a fictitious drug, Drug #0506. Navigation through this non‐linear PBL enabled student exploration using hyperlinked hotspots and buttons, where students selected one of two patients (Figure 2A). After selection of their patient, students had another branching opportunity, whereby they selected to administer Drug #0506 either intravenously or orally (Figure 2B), although selection of the patient and route of administration could be changed through backwards navigation. After this, students then determined dosages (Figure 2C,D) and were then prompted to calculate the therapeutic window, concentration maximum (*C*_max_), time to reach the maximum concentration (*T*_max_), volume of distribution (*V*_d_), half‐life (*T*_1/2_) and bioavailability (*F*) based on their selection of patient and route of administration (Figure 2E,F). The formative non‐linear PBL enabled immediate feedback if students selected the incorrect answer to the calculation and enabled another attempt via backwards navigating “try again” buttons (Figure 2F,G). When a student correctly identified the answer (Figure 2H), students could elect to return to the initial patient selection (Figure 2A) or calculate other pharmacokinetic parameters for their selected patient (Figure 2E).

To evaluate student perceptions on the use of formative non‐linear PBL assessments, an online questionnaire was administered using a Likert scale format. The questionnaire was distributed via Microsoft Forms to students who had completed the formative non‐linear PBL and contained statements relating to the non‐linear PBL assessment, with respondents being asked to indicate their level of agreement with each statement on a five‐point Likert scale ranging from “strongly agree” to “strongly disagree”. Participation was voluntary, with the questionnaire made available to students enrolled on a third‐year undergraduate pharmacology module during the 2020/2021‐2022/2023 academic years. The response rate was 15.5%, with 31 of 200 invited students responding to the online questionnaire.

## 3 RESULTS

**FIGURE 3:**
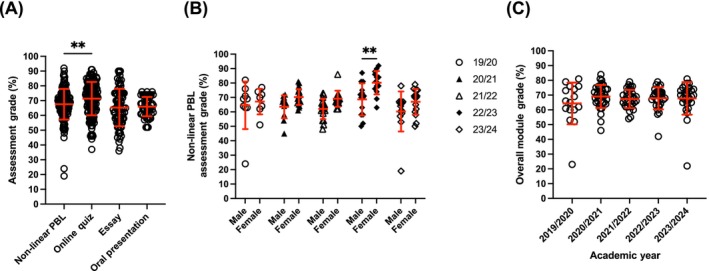
Student grades within a second‐year undergraduate pharmacology module using non‐linear problem‐based learning (PBL) summative assessments (A) grades achieved by students across four assessment techniques over 5 years for students enrolled on a second‐year undergraduate module employing non‐linear PBL assessments. **P < 0.01. Online quiz, n = 141, essay, n = 83, oral presentation, n = 56, non‐linear PBL, n = 140. (B) Grades achieved by male, n = 66, and female, n = 75, and female students for non‐linear PBL assessments across five academic years. (C) Overall module grades of students enrolled on the second‐year undergraduate module across five academic years. No statistical significance was observed across year groups. 2019/2020, n = 16; 2020/2021, n = 36; 2021/2022, n = 32; 2022/2023, n = 29; 2023/2024, n = 28. Error bars represent the mean ± standard deviation.

We observed that online quiz assessment grades were significantly higher (n = 141, mean 71.4 ± 11.3%, range 37‐91%) than non‐linear PBL assessments (n = 140, mean 67.6 ± 10.4%, range 19‐92%, *P* = 0.002; Figure 3A). However, non‐linear PBL assessments showed no significant difference in grades achieved when compared to essays (n = 83, mean 65.3 ± 12.7%, range 36‐90%; Figure 3A) and oral presentation assessments (n = 56, mean 66.0 ± 6.5%, range 52‐76%; Figure 3A).

We observed that female student grades for non‐linear PBL assessments were significantly higher in the 2022/2023 academic year (n = 14, mean 80.2 ± 8.1%, range 63‐92%) than males (*n* = 14, mean 68.6 ± 11.2%, range 50‐87%, *P* = 0.006; Figure 3B) when zero scores for no submissions were omitted from analysis. In four of the five academic years, non‐PBL assessments showed no significant difference in grades achieved between male and female students (*P* > 0.05; Figure 3B). Overall module grades showed no significant difference across the five academic years (*P* > 0.05; Figure 3C). Furthermore, the average module grades across five academic years were 67.7 ± 9.0% (range 22‐84%).

Qualitative feedback from student e‐mail correspondence and institutional module feedback on the use of summative non‐linear PBL included:

- “The [non‐linear PBL], whilst challenging, was so interesting and fun”
- “Thank you for your massive efforts in making the interactive [non‐linear PBL] and making learning so exciting – I felt like a real‐life detective it was great!”
- “If I'm honest, it almost felt like a game so thank you again for the effort you put in to making it.”
- “I had really found the [non‐linear PBL] case study AMAZING! I thought it was really great to have that interaction”.
- “I've been working in A&E since lockdown as a healthcare support worker and the [non‐linear PBL] content was so relevant and has really made me understand more about the blood gas testing, urine testing, blood testing that I see every shift and has really opened my eyes to the importance of such routine tests!”

**FIGURE 4:**
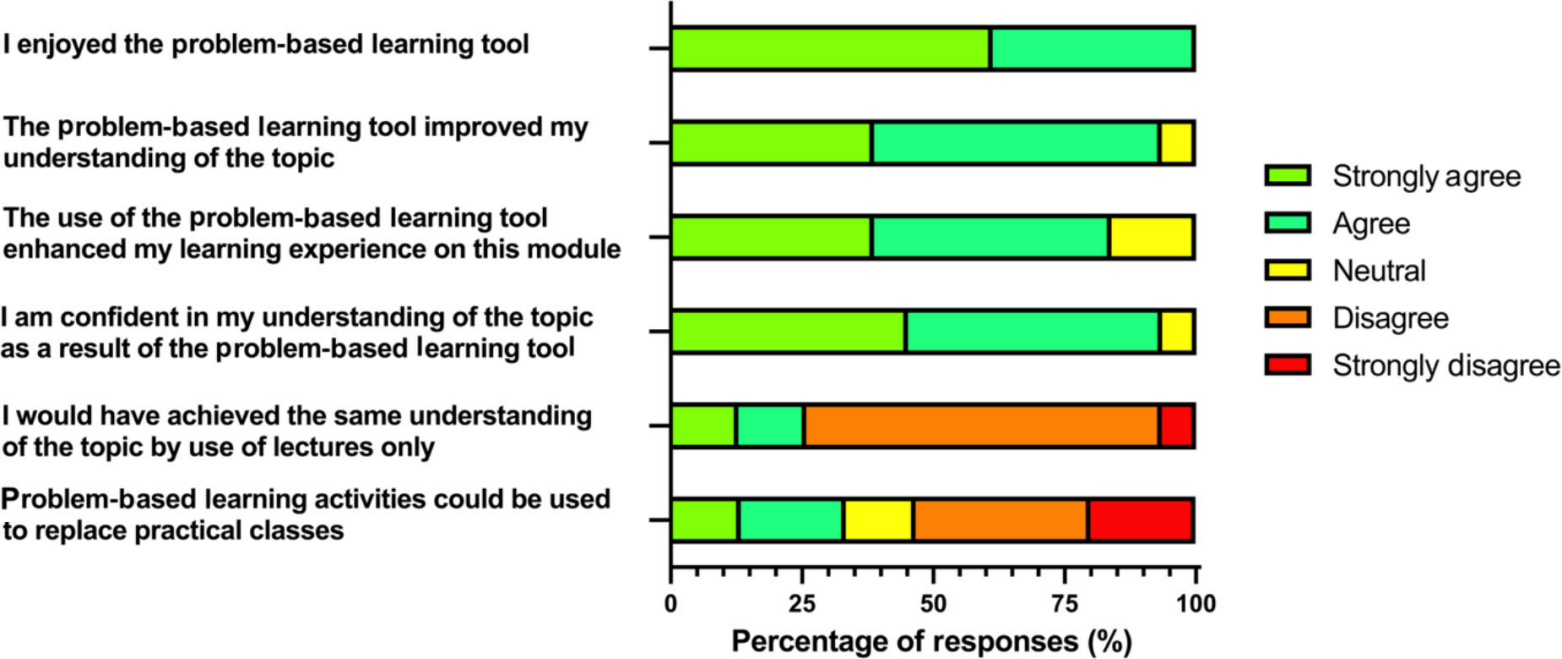
Likert scale analysis of student feedback on use of non‐linear problem‐based learning (PBL) formative assessments. Undergraduate students enrolled on a third‐year pharmacology module were asked how much they agreed or disagreed with each statement following the use of non‐linear PBL formative assessment based on pharmacokinetic calculations. N = 31.

Student feedback from use of formative non‐linear PBL assessments demonstrated that all students (n = 31) agreed or strongly agreed that they “enjoyed the Problem Based Learning tool” (Figure 4), 94% (n = 29) of respondents agreed or strongly agreed that non‐linear PBL improved their understanding and 84% (n = 26) agreed or strongly agreed that the non‐linear PBL assessment enhanced their learning experience (Figure 4). The formative non‐linear PBL assessments also improved student confidence, with 94% (n = 29) of respondents either agreeing or strongly agreeing with the statement “I am confident in my understanding of the topic as a result of the problem‐based learning tool” (Figure 4). While 26% of students (n = 8) felt they would have achieved the same understanding of the topic from lectures only, 74% (n = 23) of students either strongly disagreed or disagreed that they would have achieved the same understanding from lectures only. Additionally, 53% (n = 16) disagreed or strongly disagreed that “problem‐based learning activities could be used to replace practical classes” (Figure 4).

## 4 DISCUSSION

In this study, we have demonstrated the use of non‐linear slide decks for administering individualized PBL‐based student assessments. We demonstrate that this is a robust pedagogical approach, with achieved grades comparable to conventional assessment techniques, while providing students with an enjoyable educational experience.

We observed that student grades for non‐linear PBL are comparable to conventional essay and oral presentation assessments, with grades achieved for online quizzes being significantly higher than these assessment techniques. This was unsurprising given that increased grades in assessments using MCQs or SAQs are well documented.^32^, ^33^, ^34^

In four of the five academic years included in this analysis, we observed no difference in non‐linear PBL grades between male and female students. However, female students performed better within non‐linear PBL assessment than their male counterparts during the 2022/2023 academic year. Of note, zero scores due to non‐submission of assessments were omitted from the analysis of assessment grades and grades achieved by gender, but when zero scores are included in the analysis, no difference in the grades achieved between male and female students was observed in this academic year. These findings suggest that non‐linear PBL in pharmacology education is an effective methodology regardless of gender, akin to traditional PBL.^14^, ^15^

While non‐linear PBL was initially developed in response to COVID‐19 restrictions, the approach has remained integral since its introduction. Over five academic years, we observed consistent grades across five academic years, with the average module grade across this time corresponding with an upper second class (2.1) honours classification. This is in line with the average grades in UK higher education.^35^

Student feedback on the use of non‐linear PBL was seen to be overwhelmingly positive, which aligns with previous studies on student perceptions of PBL. In a scoping review of PBL by Trullàs et al,^5^ 85% of studies (51/60) showed that PBL was generally well received when compared with 3% (2/60) with negative satisfaction scores. Additionally, qualitative feedback from students on non‐linear PBL said that it “felt like a game” with gamification within educational settings having the potential to increase student engagement and motivation, as reviewed by Li et al.^36^ Furthermore, qualitative feedback suggested that non‐linear PBL activities enabled students to apply theory/knowledge to practical and clinical situations. PBL activities are reported to improve the development of critical thinking skills in comparison to lecture‐based content,^37^ and the non‐linear approach helps to actively engage students in decision making and understanding the consequences of those decisions.^24^

Non‐linear PBL methodology, therefore, does not significantly improve the grades achieved by students but can be used to promote student engagement, improve the student experience and improve student confidence in lecture content.

An important limitation is that student feedback must be interpreted cautiously, given the low participation rates and the potential for response bias. While online questionnaires can bypass bottlenecks in data collection, entry and analysis, this approach is known to have lower response rates than on‐paper surveys.^38^ Furthermore, online questionnaires demonstrate differences in the likelihood of responses among students, with female students more likely to respond than males and high performance positively linked to response likelihood, as reviewed by Adams and Umbach.^39^ In‐class, paper‐based questionnaires could be utilized in future to reduce these issues, improve response rates and provide a more comprehensive view of student perceptions of non‐linear PBL.

The use of non‐linear PBL does have its challenges. While non‐linear PBL approaches may also be useful in addressing recommended curricula, such the British Pharmacological Society's Undergraduate Pharmacology Core Curriculum, which states that pharmacology graduates should have the ability “to apply their skills in a real world setting”,^31^ the development of non‐linear PBL assessments is significantly more complex than conventional assessments, requiring production of extensive slide decks to facilitate student choices. For example, in this study, the summative non‐linear PBL had 86 slides while the formative non‐linear PBL had 120 slides. However, once created, minor alterations can be made to accommodate students completing reassessment periods to prevent duplication of assessments.

Educators also need to take required steps to embed these slide decks within the VLE, requiring additional digital competencies. With the increasing availability of artificial intelligence, educators must take steps to ensure that summative non‐linear slide decks cannot simply be downloaded from the VLE and uploaded to these tools. For example, cloud‐based file sharing systems now allow for “can view but not download” settings, which will allow students to view files without the ability to download files and upload elsewhere. This approach also prevents the sharing of assessments with their peers and ensures assessment validity year‐on‐year.

Despite these challenges, non‐linear PBL provides students with an assessment methodology which they receive positively and enables integration of theory and practice through the application of knowledge and skills, with grades achieved comparable to conventional assessment methodologies.

## AUTHOR CONTRIBUTIONS

**Wendy R. Francis:** Writing—original draft; writing—review and editing; visualization. **Nia A. Davies:** Conceptualization; investigation. **Aidan Seeley:** Conceptualization; methodology; formal analysis; investigation; writing—original draft; writing—review and editing; visualization.

## CONFLICT OF INTEREST STATEMENT

The authors declare no conflict of interest with the contents of this article.

## Data Availability

The data that support the findings of this study are available from the corresponding author upon reasonable request.
